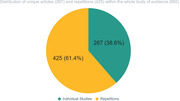# Redundancy in systematic research of exercise interventions for older adults with cognitive impairment: Is more better?

**DOI:** 10.1002/alz.095504

**Published:** 2025-01-09

**Authors:** Julie D Ries, Claudia De Santis, Kaoutar Ouabicha, Mahederemariam Bayleyegn Dagne, Pallavi Sood, Patricia C Heyn

**Affiliations:** ^1^ Center for Optimal Aging, Marymount University, Arlington, VA USA; ^2^ Faculty of Medecine, Pharmacy & Dentistry of Fes, Fes, Fes‐Meknès Morocco

## Abstract

**Background:**

Exercise as an intervention to impact cognition in older adults with mild cognitive impairment and dementia is a well‐studied phenomenon. Recent proliferation of synthesis studies on this topic might be perceived as a positive contribution to the science of exercise as medicine, but research redundancy (defined here as multiple overlapping review studies utilizing the same randomized controlled trials [RCTs]) is not only unnecessary but can be harmful. Redundancy can create research waste and be detrimental to the ability to draw confident conclusions from the evidence. The purposes of this meta‐research are to (1) evaluate redundant representation of RCTs across published meta‐analyses (MAs) on the effects of exercise on older adults with cognitive impairment, and (2) discuss implications for interpretation of the evidence.

**Method:**

This study was borne of a living evidence synthesis project, following standard synthesis methodology including PROSPERO registration, PRISMA guidelines, librarian‐assisted search strategy using multiple databases, and recurrent searches in 2015, 2018, 2020, and 2023.

**Result:**

Thirty‐eight MAs met our inclusion criteria, referencing a total of 692 RCTs. Of the 692 studies cited in the MAs, only 267 (38.6%) are unique studies. One‐hundred‐forty‐nine of those unique studies were cited in only one MA, and 118 were cited in anywhere between 2‐17 different MAs. Thus, 425 (61.4%) of the total 692 RCTs recognized across the 38 MAs were redundant. Categorized by the number of MAs in which a study was represented, 37 RCTs (13.9%) were included in 2 MAs, 48 (18%) were represented in 3‐5 MAs, 26 (9.7%) were in 6‐10 MAs, and 7 of the unique studies (2.6%) were represented in 11‐17 MAs.

**Conclusion:**

The impact of exercise on cognition of older adults with cognitive impairment is an important topic being extensively researched. While replication is a common practice in research, excessive redundancy in research does not provide new contributions and may be wasteful. Additionally, there are negative implications for meaningful interpretation of evidence when there is significant redundancy across publications. Efforts to reduce research waste might include rigorous research protocol registration requirements and the use of artificial intelligence to aid in monitoring protocols and publications.